# miRNAs and viroids utilize common strategies in genetic signal transfer

**DOI:** 10.3389/fnmol.2014.00010

**Published:** 2014-02-10

**Authors:** James M. Hill, Yuhai Zhao, Surjyadipta Bhattacharjee, Walter J. Lukiw

**Affiliations:** ^1^LSU Neuroscience Center, Louisiana State University Health Sciences Center, Louisiana State UniversityNew Orleans, LA, USA; ^2^Department of Ophthalmology, Louisiana State University Health Sciences Center, Louisiana State UniversityNew Orleans, LA, USA; ^3^Department of Microbiology, Louisiana State University Health Sciences Center, Louisiana State UniversityNew Orleans, LA, USA; ^4^Department of Neurology, Louisiana State University Health Sciences Center, Louisiana State UniversityNew Orleans, LA, USA

**Keywords:** micro RNA (miRNA), viroids, RNAseIII mediated cleavage, small non-coding RNAs, viroid-specific RNA (vsRNA), PSTV, single stranded RNA, genetic evolution

Micro RNAs (miRNAs) constitute an 18–25 nucleotide (nt), highly conserved, non-coding, single stranded RNA (ssRNA) family that are the smallest known carriers of highly selective genetic regulatory information in plants and animals (Lukiw et al., 1992, 2008, 2012; Ambros, 2004; Lukiw, 2007, 2012a; Mehler and Mattick, 2007; Bartel, 2009; Guo et al., 2010; Taft et al., 2010; Witkos et al., 2011). Similarly, viroids are non-coding, un-encapsulated, autonomously infectious circular ssRNA plant pathogens ranging in size from 246 to 401 nt and, possessing the highest *in vivo* mutation rate among all known nucleic acids, are the smallest known pathogens in all of biology (Diener, 2003; Wang and Ding, 2010). Analogous to mature miRNAs, viroid-mediated biological actions and pathogenic activities are associated with the appearance of small viroid-specific ssRNA (vsRNA), 21–24 nt in size, processed by Dicer-like proteins from a dsRNA pre-viroid precursor (Diener, 1991, 2003; Arteaga-Vazquez et al., 2006; Ding, 2009; Adams and Carstens, 2012; Hammann and Steger, 2012). While miRNAs regulate messenger RNA (mRNA) translation and decay and hence gene expression, viroids are the smallest infectious nucleic acids known that can self-replicate and transmit disease. Importantly, viroids are not only of evolutionary, genetic, and biological interest but are also of agricultural and economic concern since viroid infections reduce the yield of many important food crops worldwide. These include the developmental stunting of the common potato plant *Solanum tuberosum*, and pomaceous fruit trees which produce the common apple *Malus domestica* (Diener, 2003; Sano et al., 2010; Wang and Ding, 2010).

**Figure 1:**
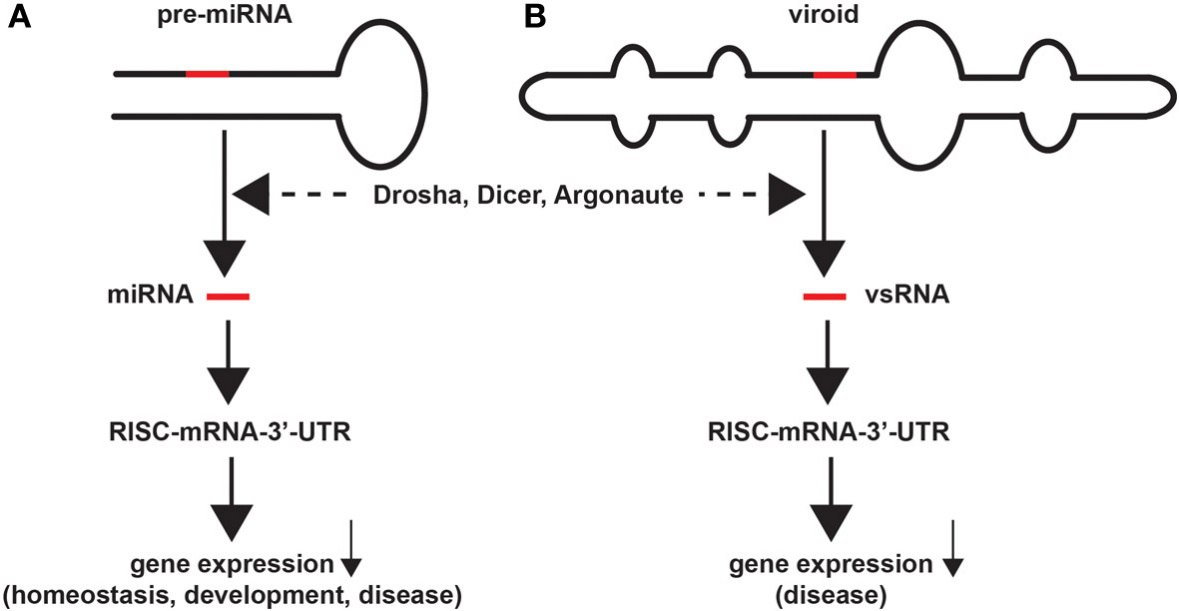
**Similarities in miRNA and viroid structure and function**. Highly schematicized figure underscoring remarkable similarities in the structure and function of miRNA and viroids; **(A)** a typical 75–110 nucleotide (nt) primary micro RNA (pri-miRNA) “hairpin” containing an endogenous 18–25 nt miRNA that yields a mature miRNA (red bar) after Drosha- and Dicer-mediated excision and processing; the mature miRNA next associates with a cytoplasmic RNA-induced silencing complex (RISC) and a target mRNA-3′-UTR to degrade and down-regulate expression of that target mRNA, with subsequent effects on the expression of genes involved in homeostasis, development and disease; **(B)** similarly a ~246–401 nt closed circular viroid, also containing extensive intra-strand base pairings and stem-loop structure(s), typically contains a 21–24 nt viroid-specific RNA (vsRNA; red bar) that after host Drosha/Dicer-based processing yields a mature vsRNA; as is the case for miRNAs this vsRNA subsequently targets the RISC and mRNA-3′-UTR complex, down-regulating gene expression to induce disease in plants (Krol and Krzyzosiak, 2006; Ritchie et al., 2007; Ding, 2009; Triboulet and Gregory, 2010). In both cases larger miRNA or vsRNA precursors are processed by an RNase III of the family of Dicer-like proteins to generate smaller ssRNA species; these sizes are similar to endogenous small interfering RNA (as mature vsRNA or miRNA) to alter the viroid-dependent gene expression in the host plant by viroids, or of miRNA-mRNA processing in animal species including humans (Arteaga-Vazquez et al., 2006; Krol and Krzyzosiak, 2006; Ritchie et al., 2007; Ding, 2009; Kosik, 2009; Triboulet and Gregory, 2010; Hammann and Steger, 2012; Navarro et al., 2012). While naked, mature RNAs such as miRNAs and vsRNAs have relatively short half-lives *in vitro* (for example human neuronal miRNAs appear to be highly labile; Sethi and Lukiw, 2009; Krol et al., 2010), stabilities may be greatly extended by single- or double-stranded RNA-binding proteins, by complex secondary structures, by RNA circularization, by containment in protease- and RNase-resistant vesicles, or by combinations of these and other factors (Chen and Shyu, 1995; Cui et al., 2005; Sethi and Lukiw, 2009; Krol et al., 2010). Interestingly, viroids, at about twice the size of typical miRNAs, are the smallest known self-replicating pathogens of all living species, and both the plant and animal kingdoms have adopted similar ssRNA strategies to store and transmit only the most essential genetic regulatory information in the propagation of either homeostatic of pathological signals. The potential for interaction between vsRNA and miRNA in their hosts, if any, amongst diverse plant and animal species is currently not known.

We summarize here that from evolutionary, structural, and mechanistic perspectives, at least 18 interdependent lines of evidence currently support the idea that miRNAs and viroids have common and distinguishing genetic features, and share overlapping regulatory and pathogenic mechanisms with intrinsic potential to promote systemic disease. These include the observations that: (i) each miRNA or viroid dsRNA precursor is generated, utilizing exclusively, host-encoded nuclear transcription components and mechanisms (Diener, 2003; Ritchie et al., 2007; Sethi and Lukiw, 2009; Wang and Ding, 2010; Adams and Carstens, 2012); (ii) from the primary parent-miRNA or viroid-precursor RNA PolII-generated transcript, intra-molecular hydrogen bonding generates a highly structured dsRNA “intermediate” precursor (Ritchie et al., 2007; Ding, 2009; Lukiw, 2012b; Navarro et al., 2012; Perkel, 2013); (iii) secondary structures are at least as important as primary sequences in infectivity and pathogenicity (Rocheleau and Pelchat, 2006; Sethi and Lukiw, 2009; Perkel, 2013); (iv) mature miRNAs or viroids are always excised from a larger, highly structured dsRNA precursor (Figure 1; Diener, 2003; Ambros, 2004; Ritchie et al., 2007; Bartel, 2009; Ding, 2009; Hammann and Steger, 2012); (v) the RNaseIII-like enzymes like Drosha and Dicer in concert process dsRNA precursors into small 18–25 nt mature ssRNAs (i.e., mature miRNA or vsRNA) (Hedges, 2002; Diener, 2003; Ambros, 2004; Bartel, 2009; Ding, 2009; Hammann and Steger, 2012); (vi) mature miRNA or vsRNA are transported out of the nucleus via Exportin-5 or highly-related transport mechanisms (Diener, 2003; Krol and Krzyzosiak, 2006); (vii) mature miRNAs or vsRNAs both appear, in part, to direct RNA-induced silencing complexes to degrade target mRNAs (Ambros, 2004; Mehler and Mattick, 2007; Bartel, 2009; Ding, 2009; Hammann and Steger, 2012; Lukiw, 2012b); (viii) both miRNA and viroid ssRNA and their precursors have evolved complex secondary and/or tertiary structures designed to minimize their own degradation (Chen and Shyu, 1995; Mehler and Mattick, 2007; Navarro et al., 2012); (ix) their small size (~18–25 nt) may protect mature miRNA or vsRNA against further cleavage by Drosha, Dicer, and other RNAseIII enzyme systems (Bartel, 2009; Ding, 2009; Diermann et al., 2010; Hammann and Steger, 2012); (x) unique ssRNA sequences of 22 nt, a common size for miRNAs and viroids, would occur only once per human genome which may have bearing on why these ssRNAs are highly enriched in particular cell types (Mehler and Mattick, 2007; Ding, 2009); (xi) neither miRNA or viroids encode proteins; their biological effects are accomplished only through highly selective RNA-RNA interactions (Rocheleau and Pelchat, 2006; Saetrom et al., 2006; Sano et al., 2010; Perkel, 2013); (xii) both mature miRNAs and vsRNA are highly soluble and mobile genetic-information carrying elements (Diener, 2003; Cui et al., 2005; Ding, 2009; Lukiw et al., 2010, 2012; Lukiw, 2012c); (xiii) mature ssRNAs are highly abundant exterior to the cells from which they originate, including high abundance in circulatory fluids like the cerebrospinal fluid (CSF) and blood serum (Ding, 2009; Alexandrov et al., 2012; Lukiw et al., 2012); (xiv) vsRNAs or miRNAs are abundant and remarkably bioactive in all species so far examined (Diener, 2003; Ambros, 2004; Wang and Ding, 2010; Lukiw, 2012b); (xv) both mature miRNAs and viroids, but not their precursors, are potentially pathogenic, and capable of inducing disease in the same cells, tissues, and species in which they were originally generated (Saetrom et al., 2006; Lukiw and Pogue, 2007; Pogue et al., 2010); (xvi) these diseases range from stunting diseases of plants to cancers and neurodegenerative disorders of *Homo sapiens*, representing a highly similar RNA-based pathological disease mechanism conserved over at least 1.5 × 10^9^ years (Diener, 2003; Lukiw et al., 2008; Ding, 2009; Wang and Ding, 2010); (xvii) the genomes and genetic mechanisms of both DNA- and RNA-based “helper” viruses may enhance the pathogenicity of both miRNA- and viroid-mediated infections (Hill et al., 2009; Pogue et al., 2010; Wang and Ding, 2010; Navarro et al., 2012; Ball et al., 2013), and (xviii) both miRNA and viroid nucleic acid sequences continue to rapidly evolve, impacting highly specific genotypic and phenotypic aspects of development, homeostasis and disease in multiple species (Diener, 2003; Kosik, 2009; Wang and Ding, 2010).

These intriguing similarities between the structure and function of miRNAs and viroids underscore the idea that once nature has found and tested a successful molecular design for information transfer it is highly preserved, and this design is used repeatedly over evolution in diverse biological applications across multiple forms of life. Indeed, much of viroid biology appears to be reiterated in the genetic mechanism of miRNA actions throughout the plant and animal kingdoms. These commonalities are based in the intrinsic molecular-genetic mechanism of miRNA and viroid RNA sequence structure and complementarity-mediated ssRNA-mRNA recognition based on hydrogen bonding. Intriguingly, small non-coding ssRNA, miRNA, and viroid ribonucleotide sequences contain fingerprints for conservation across multiple species, and these fingerprints represent some of the most highly conserved nucleic acid sequences known (Arteaga-Vazquez et al., 2006; Shi et al., 2012). Interestingly, it has been recently demonstrated that infection of human brain cells in primary culture with a particularly virulent strain of the dsDNA herpes simplex-1 virus (HSV-1) induces, and then utilizes a host-specific pro-inflammatory miRNA-146a to support and propagate its invasiveness and successful infection (Hill et al., 2009; Lukiw et al., 2010; Ball et al., 2013). It will certainly be interesting to understand if other RNA- or DNA-based “helper viruses” promote or intensify the actions of miRNA or viroids *in vivo*, if miRNA effects can be moderated by vsRNA or other ssRNA or dsRNA, if miRNA and viroid activities are equally affected by the presence of natural circular RNAs (circRNAs), and what potential roles other environmental and epigenetic factors may play in miRNA- or vsRNA-mediated gene activity and pathogenicity as disease moderators in the CNS (Krol and Krzyzosiak, 2006; Lukiw, 2012a, 2013; Hansen et al., 2013; Memczak et al., 2013). Importantly, the potential for spreading of miRNA and viroid information-carrying signals from cell to cell, tissue to tissue and perhaps even between species has a enormous bearing on our understanding on the complex genetic interactions between diverse forms of life in both the plant and animal kingdoms, and their potential for symbiotic exchanges of genetic information in natural environments (Orgel, 1968; Hedges, 2002; Arteaga-Vazquez et al., 2006; Ding, 2009; Wang and Ding, 2010; Alexandrov et al., 2012; Hammann and Steger, 2012; Bhattacharjee and Lukiw, 2013; Sarkies and Miska, 2013; Perkel, 2013).
